# Breast Cancer Patients at Increased Risk of Developing Type II Endometrial Cancer: Relative and Absolute Risk Estimation and Implications for Counseling

**DOI:** 10.7759/cureus.12981

**Published:** 2021-01-29

**Authors:** Sara Portela, Aimee Cunningham, Alexandros Laios, Richard Hutson, Georgios Theophilou

**Affiliations:** 1 Gynaecological Oncology, Leeds Teaching Hospitals NHS Trust, Leeds, GBR

**Keywords:** endometrial carcinomas, tamoxifen, breast cancer management, hysterectomy

## Abstract

Introduction

Breast cancer (BC) is a recognized risk factor for endometrial cancer (EC). Emerging literature indicates that it confers a higher risk of type II EC (T2EC) than type I EC (T1EC). Although some surgeons offer a prophylactic hysterectomy to BC patients referred for risk-reducing bilateral salpingo-oophorectomy, insufficient evidence prevents this from being the standard practice. We aimed to quantify their absolute risk and relative risk (RR) of developing both EC subtypes and identify a higher-risk group that could be considered for prophylactic hysterectomy.

Methodology

This retrospective service evaluation compared patients diagnosed with BC between 2008 and 2014, who subsequently developed EC within 10 years to those who did not. Absolute risk and RR were calculated using the numbers of regional BC and EC cases within this group, alongside 2009 UK female population and EC incidence statistics. Binary logistic regression generated adjusted odds ratios (ORs) for patient- and disease-specific variables.

Results

A total of 45 BC patients developed EC, 24 had T1EC and 21 had T2EC. Their RR of developing EC was greater than that of the general population (RR: 12.44, p < 0.0001). Notably, this was higher for T2EC (RR: 33.96, p < 0.001) than T1EC (RR: 8.63, p < 0.0001). Nonetheless, the absolute risk remained low. Tamoxifen exposure was significantly more prevalent among T2EC patients (adjusted OR: 79.61, p = 0.003). Increased age at BC diagnosis was associated with T1EC (adjusted OR: 1.10, p = 0.043) and T2EC (adjusted OR: 1.13, p = 0.03). Neither smoking status nor family history of BC was significantly associated with any outcome.

Conclusion

Women with BC were more likely to develop T2EC than T1EC, and although the absolute risk was low, the cumulative risk was substantial enough to warrant vigilance. Tamoxifen exposure was significantly predictive of EC, particularly T2EC, and might facilitate risk estimation. Older women at BC diagnosis who receive tamoxifen treatment should be screened and closely monitored for EC. However, given the limitations of normal screening methods for the detection of T2EC, counseling for a prophylactic hysterectomy should also be considered. Clarification of the menopausal status will help make more meaningful recommendations.

## Introduction

Endometrial cancer (EC) is the fifth most common female malignancy in developed countries. The traditional classification of EC into type I (T1EC) and type II (T2EC) reflects their different clinicopathological characteristics, including pathogenesis and clinical outcomes [1]. T2EC is recognized as having a worse outcome due to late presentation and increased tendency to metastasize. Nevertheless, increasing evidence suggests that they may share several etiological factors with similar magnitude [2].

Breast cancer (BC) is a recognized risk factor for EC, particularly T1EC [3-5]. Emerging literature now indicates women with BC may be more likely to develop T2EC than T1EC. Tamoxifen is a plausible risk factor for this [6,7]. This suggests that, in contrast to previous convictions, the etiology of T2EC may not be completely estrogen independent.

Primary BC clinical practice guidelines do not include prophylactic measures against EC. At Leeds Teaching Hospitals, high-risk BC patients (i.e., strong family history of breast and/or ovarian cancer, previous BC history, *BRCA* mutation) are referred to the gynecology oncology team for consideration of risk-reducing bilateral salpingo-oophorectomy. Despite not being the standard practice, concomitant hysterectomy is sometimes offered to protect against EC. The national and global discrepancies in practice may mean women are unknowingly missing out on life-saving surgery. Therefore, more evidence is required for patients and clinicians to make informed risk-benefit decisions about prophylactic measures.

To address the above, we aimed to clarify the role of prophylactic hysterectomies in the treatment of BC patients by determining their absolute risk and relative risk (RR), compared to the general population, of developing each clinicopathologic EC type. Equally, we aimed to identify a higher-risk group that could be considered for prophylactic hysterectomy.

## Materials and methods

Study design

This service evaluation compared patients diagnosed with BC between 2008 and 2014, who subsequently developed EC within 10 years to those who did not. Patient- and disease-specific variables associated with EC subtypes were examined. As a service evaluation, this study did not require any ethical approval.

Participants

All women diagnosed with BC between January 2008 and December 2014 were identified; those diagnosed after 2014 were excluded to allow for an appropriate follow-up period of at least four years in which EC could develop. As per the International Classification of Diseases for Oncology, EC and BC were defined according to primary site codes C54.1 to C55 and C50 and D05, respectively.

All women who had developed EC between January 2008 and December 2014 following a BC diagnosis were identified. They were compared to BC patients of a similar cohort size who did not subsequently develop EC, selected through simple random sampling using the Research Randomizer software® [8]. Of these, those with a past medical history of EC, concurrent malignancy, or with breast tumor morphology not identified in the other cohort (i.e., all but ductal carcinoma, lobular carcinoma, unspecified breast carcinoma) were excluded.

Variables

The patient-related variables collected included date of birth, smoking history, family history of BC and EC, menopausal status at the time of EC diagnosis, and tamoxifen used for invasive BC treatment. Menopausal status at the time of BC diagnosis could not be verified as patients were often treated for their BC at different trusts, and therefore, their medical records at the time were not available. Positive family history was defined as the occurrence of EC in a first-degree relative. The disease-related variables collected for both BC and EC included date of diagnosis, tumor morphology, and tumor grade. Should BC recur prior to the development of EC, then the disease-related variables were collected for the first tumor.

Data sources/measurements

Preexisting data were extracted from electronic and paper medical records. Smoking history was recorded as a binary variable (never smoked versus current or previous smoker) as was tamoxifen exposure (no exposure versus exposure). EC was categorized as T1EC or T2EC according to the clinicopathological classification [1].

Quantitative variables

The incidence of EC in the UK in 2009, stratified by morphology, was obtained from the National Cancer Registration and Analysis Service [9]. This was re-categorized into T1EC and T2EC to determine the UK incidence of each type in 2009. Female 2009 UK population statistics were obtained from the Office for National Statistics and used to calculate the absolute risks [10].

Statistical methods

The absolute and RR of developing T1EC and T2EC were calculated for women treated for BC in Leeds compared to the general UK population. Average number of cases per year was calculated to account for the difference in observation periods for the BC and EC cohort (10 years) compared to the BC-only cohort (six years). Odds ratios (OR), both adjusted and unadjusted, with 95% confidence interval (CI) were calculated for all nominal variables to identify associations with specific EC subtypes. Descriptive statistics and binary logistic regression were performed using SPSS version 26 (IBM, Armonk, NY, USA). Missing data were handled by pairwise deletion. Statistical significance was set at p < 0.05.

## Results

Participants

**Figure 1:**
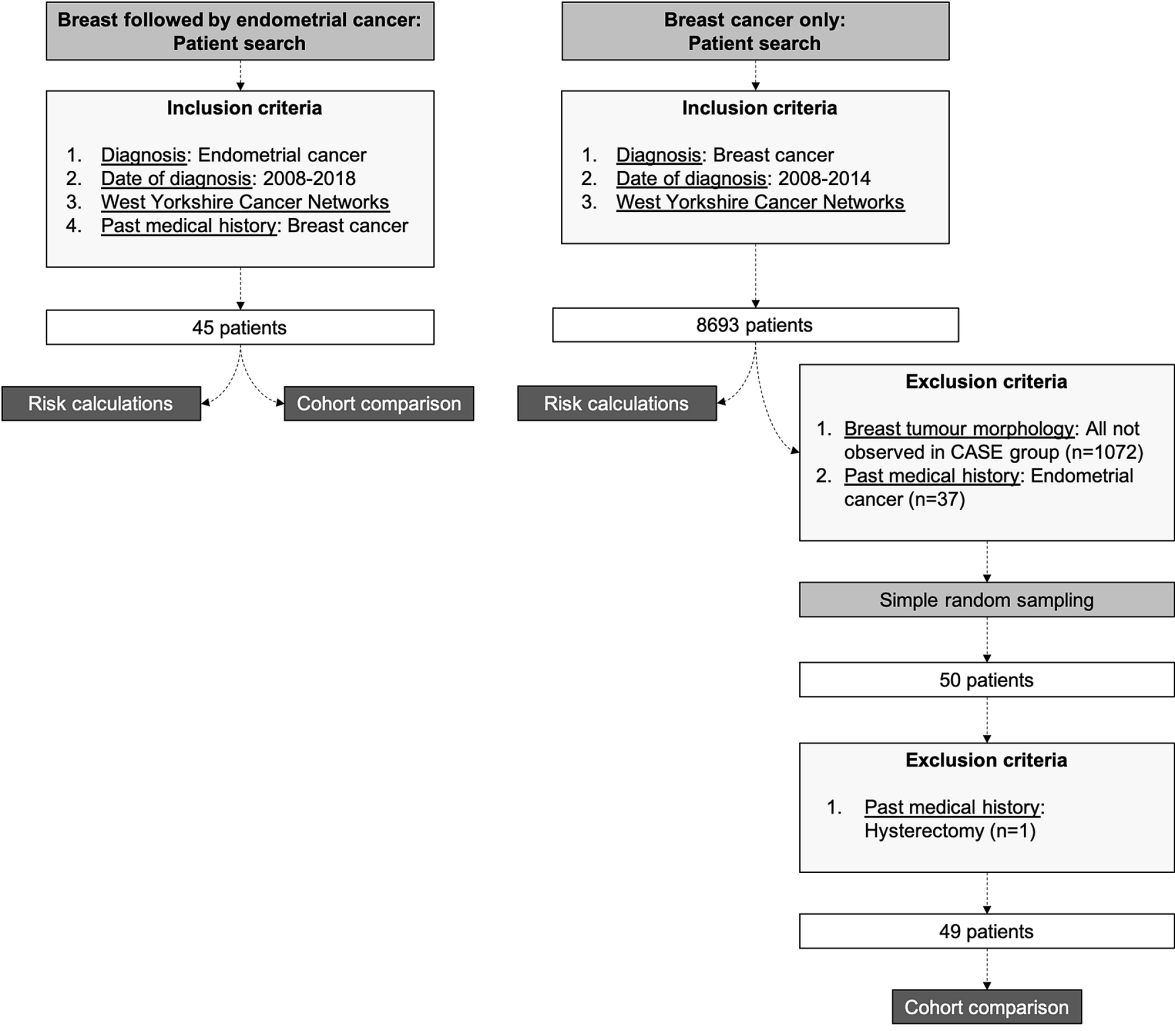
Patient selection flowchart with inclusion and exclusion criteria.

A total of 45 patients with a history of BC were diagnosed with EC between 2008 and 2018. Of the 8,693 women diagnosed with BC between 2008 and 2014, 50 were selected through random sampling [8]. One patient was excluded from case-control analysis due to having undergone a hysterectomy (Figure 1).

Descriptive data

A total of 24 women developed T1EC, including endometrioid (n = 23) and mucinous (n = 1) adenocarcinomas. A total of 21 women developed T2EC, including serous adenocarcinomas (n = 7), carcinosarcomas (n = 6), papillary carcinomas (n = 2), endometrial stromal sarcomas (n = 2), sarcoma (n = 1), mixed cell adenocarcinoma (n = 1), adenosarcoma (n = 1), and a müllerian mixed malignant tumor (n = 1). Their BC morphology included ductal carcinoma (n = 33), of which only one was intraductal/non-invasive, lobular carcinoma (n = 4) and unspecified (n = 8).

**Figure 2:**
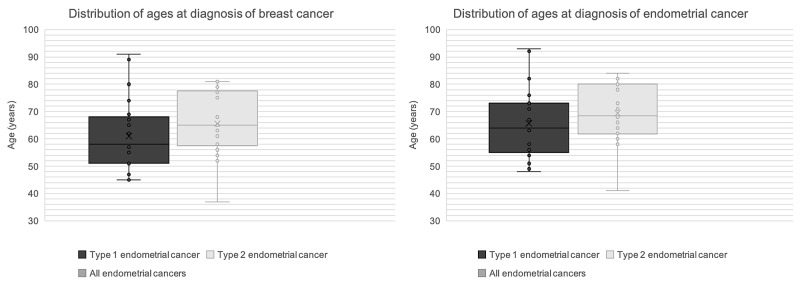
Distribution of ages at diagnosis of breast and endometrial cancers.

The average ages at diagnosis of BC and EC were 63 and 67 years, respectively (Figure 2).

Only two patients were premenopausal at the time of EC diagnosis, one with T1EC (41 years old) and one with T2EC (49 years old). The mean time between BC and EC diagnoses was four years, ranging from 83 days to 12 years. The frequency and proportion of patient- and disease-related variables are summarized in Table 1.

**Table 1:** Demographic, morphological, and treatment characteristics by disease outcome.

Variables	Breast cancer only (n = 49)		All endometrial cancers (n = 45)		Type I endometrial cancer (n = 24)		Type II endometrial cancer (n = 21)	
	n	%	n	%	n	%	n	%
Ethnicity								
Missing	19	38.78	12	26.67	6	25.00	6	28.57
Caucasian	28	57.14	31	68.89	17	70.83	14	66.67
Pakistani	2	4.08	1	2.22	1	4.17	0	0.00
Afro-Caribbean	0	0.00	1	2.22	0	0.00	1	4.76
Breast cancer grade								
Missing	14	28.57	4	8.89	3	12.50	1	4.76
0	0	0.00	1	2.22	1	4.17	0	0.00
1	8	16.33	9	20.00	6	25.00	3	14.29
2	19	38.78	21	46.67	12	50.00	9	42.86
3	8	16.33	10	22.22	2	8.33	8	38.10
Endometrial cancer grade								
Missing	-	-	5	11.11	0	0.00	5	23.81
0	-	-	0	0.00	0	0.00	0	0.00
1	-	-	14	31.11	13	54.17	1	4.76
2	-	-	11	24.44	9	37.50	2	9.52
3	-	-	15	33.33	2	8.33	13	61.90
Tamoxifen exposure								
Missing	1	2.04	4	8.89	2	8.33	2	9.52
No	33	67.35	18	40.00	6	25.00	12	57.14
Yes	15	30.61	23	51.11	16	66.67	7	33.33
Smoking status								
Missing	16	32.65	13	28.89	9	37.50	4	19.05
Never	26	53.06	19	42.22	10	41.67	9	42.86
Past	3	6.12	12	26.67	5	20.83	7	33.33
Current	4	8.16	1	2.22	0	0.00	1	4.76
Positive family history								
Breast cancer	7	14.29	7	15.56	4	16.67	4	19.05
Endometrial cancer	2	4.08	0	0.00	0	0.00	3	14.29
Menopausal status at the time of endometrial cancer diagnosis								
Missing	-	-	1	2.22	1	4.17	0	0
Premenopausal	-	-	2	4.44	1	4.17	1	4.76
Postmenopausal	-	-	42	93.33	22	91.67	20	95.24

Main results

Unadjusted OR calculations showed a significantly greater likelihood of observing previous tamoxifen use among patients who subsequently developed EC (OR: 2.81, p = 0.02), but more specifically T1EC (OR: 5.87, p = 0.002). The observation was not statistically significant for T2EC (OR: 1.28, p = 0.66). Age at BC diagnosis significantly predicted EC development (OR: 1.22, p = 0.024). The observation was significant for both T1EC (OR: 1.19, p = 0.032) and T2EC (OR: 1.25, p = 0.02). Analysis of the family history of BC and smoking history did not generate statistically significant differences between patients who had only had BC and those who subsequently developed EC (Table 2).

**Table 2:** Unadjusted odds ratios of breast cancer patient outcomes per variable.

Variables	All endometrial cancers (n = 45)			Type I endometrial cancer (n = 24)			Type II endometrial cancer (n = 21)		
	Odds ratio	95% confidence interval	P-Value	Odds ratio	95% confidence interval	P-Value	Odds ratio	95% confidence interval	P-Value
Age at breast cancer diagnosis	1.22	1.05, 5.32	0.024	1.19	1.02,1.32	0.032	1.25	1.12, 1.38	0.02
Tamoxifen exposure	2.81	1.18, 6.69	0.02	5.87	1.92, 17.97	0.002	1.28	0.42, 3.91	0.66
Smoking status	2.54	0.85, 7.58	0.09	1.86	0.48, 7.23	0.37	3.30	0.93, 11.71	0.06
Family history of breast cancer	1.09	0.35, 3.46	0.88	1.18	0.30, 4.56	0.81	1.00	0.23, 4.40	1.00

Binary logistic regression identified patient age at BC diagnosis (OR: 1.11, p = 0.011) and tamoxifen exposure (OR: 14.63, p = 0.003) as significant predictors of EC. Increasing age was significantly associated with both T1EC (OR: 1.10, p = 0.043) and T2EC development (OR: 1.13, p = 0.026). Tamoxifen exposure was also significantly associated with developing T2EC (OR: 79.61, p = 0.003). Smoking history and family history of BC were not predictive of the development of EC or its subtypes (Table 3).

**Table 3:** Adjusted odds ratios of breast cancer patient outcomes per variable.

Variables	All endometrial cancers (n = 45)			Type I endometrial cancer (n = 24)			Type II endometrial cancer (n = 21)		
	Odds ratio	95% confidence interval	P-Value	Odds ratio	95% confidence interval	P-Value	Odds ratio	95% confidence interval	P-Value
Age at diagnosis of breast cancer	1.11	1.03, 1.21	0.011	1.10	1.00, 1.21	0.043	1.13	1.02, 1.26	0.03
Tamoxifen exposure	14.63	2.43, 87.90	0.003	5.33	0.70, 40.34	0.105	79.61	4.63, 1369.33	0.003
Smoking status	1.98	0.54, 7.24	0.300	1.83	0.41, 8.19	0.431	1.78	0.29, 11.06	0.534
Family history of breast cancer	1.32	0.27, 6.58	0.734	0.80	0.10, 6.64	0.834	3.68	0.41, 33.41	0.247

Risk analysis

Patients with a history of BC had a 12-fold risk (p < 0.0001) of developing EC compared to the general UK population, particularly T2EC (RR: 33.96, p < 0.0001) (Table 4).

**Table 4:** Absolute and relative risks of endometrial cancer and its clinicopathologic types.

Endometrial cancer type	Absolute risk		Relative risk	P-Value (95% confidence)
	General population	Study population		
All	0.00025	0.00310	12.44	<0.0001
Type 1	0.00019	0.00165	8.63	<0.0001
Type 2	0.00004	0.00145	33.96	<0.0001

Unlike the female general population in 2009 who had a 4.75-fold greater risk of developing T1EC, BC patients had a 1.14-fold higher risk of developing T2EC (0.145% absolute risk) than T1EC (0.165% absolute risk).

## Discussion

Key results

This retrospective study found that patients with a history of BC had 12 times the risk of developing EC, eight times the risk of developing T1EC, and almost 34 times the risk of developing T2EC compared to the general UK population. The absolute risk of developing T2EC was greater than that of T1EC for BC patients, which is the inverse of the trend observed in the general population. Despite this, the yearly absolute risk was only 1/322, 1/606, and 1/689 for EC, T1EC, and T2EC, respectively. Both increasing patient age at the time of BC diagnosis and tamoxifen exposure were significantly associated with the development of EC, particularly T2EC. Increasing age was also associated with T1EC. To the best of our knowledge, this is the first study to quantify the absolute risk and RR of developing the clinicopathological subtypes of EC among BC patients.

Interpretation

If BC patients are at an increased risk of EC, then a prophylactic hysterectomy can effectively protect against this risk. To assess the need for hysterectomy, the absolute risk and RR of the BC cohort should be first estimated. Few previous studies attempted to calculate standardized incidence ratios for all ECs but not the EC subtypes. Our results are concordant with previous international studies [6,11-13].

Unsurprisingly, tamoxifen exposure was a significant predictor of EC development in BC patients in both adjusted and unadjusted OR calculations, which was mainly the case with T2EC. Tamoxifen has been repeatedly linked with EC [4]. Fisher et al. estimated an annual hazard rate of 1.6/1,000 following tamoxifen use compared with 1.2/1,000 in the placebo group [14]. Despite no consensus on the pathogenic effect of tamoxifen leading to T2ECs, several studies have identified a link between exposure to this estrogen receptor modulator and this aggressive type of EC [15,16]. Nevertheless, this finding has not been consistent across all histological subtypes of T2EC [7]. In our study, we observed some inconsistency between the adjusted and unadjusted ORs, highlighting the difficulty in determining the true effect of possible risk factors. The small sample size, noticeable when comparing EC types, may have contributed to this. The length and recency of tamoxifen exposure did not form a part of this analysis. Existing literature indicates a pertinent role for the duration of tamoxifen use. A nationwide case-control study evaluating the risk and prognosis of EC after tamoxifen use determined the adjusted risk increased significantly with longer duration of tamoxifen use [17], which was confirmed by other studies [13,15,17]. The direction and magnitude of the association between BC and EC reported in these studies are in line with our results, although after adjustment of covariates, the magnitude of the effect was found to be substantially higher. Crucially, these studies have not investigated the effect of tamoxifen on the risk of developing specific types of EC, hindering corroboration of our results.

Age at BC diagnosis was found to be a significant predictor of EC and both its subtypes. The effect on risk was homogenous for all outcomes. This prediction also remains significant in the age-adjusted model. However, discrepancies in the literature regarding the relevance of age warrant further research on the topic. As BC and EC are related to several hormonal and reproductive risk factors, it is reasonable to assume that parity, early menarche, and age at first birth could be more sensitive markers in the studied association. Unfortunately, such information was not available for most of our cohort patients. However, we clearly demonstrated that for every increase in age by a decade, the risk for EC increased by more than 10%. It would have been less prudent to set an age cut-off point for offering intervention as the results may be skewed by the individual reproductive outcomes. Other studies have considered outcomes relating to the age at BC diagnosis in combination with tamoxifen use [4,18]. Jones et al. found that age did not impact EC mortality, implying it has little clinical relevance [19]. In the UK, tamoxifen is only licensed for pre/perimenopausal women; it has a greater chance of affecting the endometrium in postmenopausal women [20]. A recent study showed the odds of developing EC following exposure to tamoxifen increased four-fold for those older than 35 years old [4]. Using 35 years as a cut-off point complicates the results and leads to questioning whether tamoxifen has negative effects prior to the menopause, or whether the results are skewed by the data from the postmenopausal women in this cohort.

Menopausal status was not significantly different at the time of diagnosis of EC. We acknowledge that information about the menopausal status at the time of BC diagnosis was not available. To investigate potential effect modification by menopausal status, some studies favor the 50th birthday as a cut-off [21]. Nevertheless, we assumed that menopausal status at the time of BC diagnosis would not be a confounding factor due to the similar age distribution at diagnosis for both cancers (Figure 2).

Smoking history and family history of BC were not significantly associated with any outcome. Smoking is thought to reduce circulating endogenous estrogen, resulting in a reduced EC risk. The lack of association observed between smoking and developing EC after BC suggests the main risk mechanism in this subset of women is tamoxifen exposure rather than endogenous estrogen. Family history of any cancer appears to increase EC risk in women with prior BC, suggesting genetic risk factors for EC [22]. Nevertheless, this was not confirmed in our study, possibly due to the small sample size.

The role of prophylactic hysterectomy requires some additional comment. We found that the absolute risk of developing T1EC and T2EC in BC patients was 1/604 and 1/690 women per year, respectively. Given the age range in our BC cohort was 54 years, the cumulative risk of developing EC would be 0.089 and 0.078 for type 1 and type 2, respectively. Therefore, one would need 11 hysterectomies to detect one T1EC and almost 13 hysterectomies to detect one T2EC. Although this is an invasive procedure with possible significant complications, it has been shown to significantly improve overall and breast-specific survival in BC patients [23,24]. Premenopausal women were shown to gain the most benefit from prophylactic hysterectomy and bilateral salpingo-oophorectomy. However, a lack of consistent improvement for non-BC-related mortality indicates survival was not affected by the presence of EC in those with no surgery. In these studies, EC types were not analyzed separately, and the small sample size may have underpowered the survival impact of the higher-risk cancers. Furthermore, the postmenopausal cohort that received surgery was small, preventing the true benefits of surgery in relation to age from being determined.

Currently, regular follow-up of BC patients being treated with tamoxifen is common practice aiming to detect symptoms of uterine malignancy. However, T2ECs are notoriously asymptomatic for extensive periods, hence their detection at a later stage. Therefore, a medical review may be insufficient to detect EC occurrence. Transvaginal ultrasound effectively detects endometrial thickening, but T2ECs present with endometrial atrophy, thus potentially accounting for this method’s high false-negative rate [25-27]. Additionally, cancer surveillance contributes to patient anxiety and ill-being [28].

Limitations

Limitations of this study included the small sample size and its retrospective nature. Incomplete documentation in medical records prevented expanding the analysis to other variables, including body mass index, parity, and duration of tamoxifen use. RR calculations compared the national EC incidence in the general population to the local population; health inequalities may hinder comparability. The accuracy of this calculation may be flawed by insufficient adjustment due to lacking information regarding confounding factors, resulting in an overestimation. Nonetheless, this study provides a novel insight into the absolute risk of the clinicopathologic subtypes of EC among BC patients.

## Conclusions

We surmise that BC women are more likely to develop T2EC than T1EC and their RR is substantial enough, albeit their absolute risk is small. Older women at BC diagnosis who will receive tamoxifen treatment should be screened and closely monitored for EC. However, given the limitations of normal screening methods for the detection of T2EC, patient counseling about a prophylactic hysterectomy should also be considered.

Nevertheless, more robust evidence produced by prospective national studies is necessary to corroborate our findings and identify additional factors that reliably predict the risk of developing each subtype of EC. The absolute risk of developing EC among tamoxifen users and non-users will be an important focus of future research. Survival analyses for patients developing T1EC and T2EC after BC are crucial to better understand the impact and consequences of these outcomes. Addressing age would also be beneficial for a risk-benefit discussion, especially because impacts of surgery differ with age. This line of research would help inform clinicians’ decisions about BC treatment and enable women to regain some agency over their future health and quality of life.
